# Antimicrobial properties of *Lactobacillus* cell‐free supernatants against multidrug‐resistant urogenital pathogens

**DOI:** 10.1002/mbo3.1173

**Published:** 2021-04-09

**Authors:** Marina Scillato, Ambra Spitale, Gino Mongelli, Grete Francesca Privitera, Katia Mangano, Antonio Cianci, Stefania Stefani, Maria Santagati

**Affiliations:** ^1^ Department of Biomedical and Biotechnological Sciences, Microbiology Section University of Catania Catania Italy; ^2^ Department of Biomedical and Biotechnological Sciences Oncologic, Clinical, and General Pathology Section University of Catania Catania Italy; ^3^ Department of General Surgery and Medical‐Surgical Specialties University of Catania Catania Italy

**Keywords:** antimicrobial activity, Lactobacilli, MDR‐urogenital infection, supernatants, vaginal probiotics

## Abstract

The healthy vaginal microbiota is dominated by *Lactobacillus* spp., which provide an important critical line of defense against pathogens, as well as giving beneficial effects to the host. We characterized *L*. *gasseri* 1A‐TV, *L*. *fermentum* 18A‐TV, and *L*. *crispatus* 35A‐TV, from the vaginal microbiota of healthy premenopausal women, for their potential probiotic activities. The antimicrobial effects of the 3 strains and their combination against clinical urogenital bacteria were evaluated together with the activities of their metabolites produced by cell‐free supernatants (CFSs). Their beneficial properties in terms of ability to interfere with vaginal pathogens (co‐aggregation, adhesion to HeLa cells, biofilm formation) and antimicrobial activity mediated by CFSs were assessed against multidrug urogenital pathogens (*S*. *agalactiae*, *E*. *coli*, KPC‐producing *K*. *pneumoniae*, *S*. *aureus*, *E*. *faecium* VRE, *E*. *faecalis*, *P*. *aeruginosa*, *P*. *mirabilis*, *P*. *vulgaris*, *C*. *albicans*, *C*. *glabrata*). The *Lactobacilli* tested exhibited an extraordinary ability to interfere and co‐aggregate with urogenital pathogens, except for *Candida* spp., as well as to adhere to HeLa cells and to produce biofilm in the *Lactobacillus* combination. *Lactobacillus* CFSs and their combination revealed a strong bactericidal effect on the multidrug resistant indicator strains tested, except for *E*. *faecium* and *E*. *faecalis*. The antimicrobial activity was maintained after heat treatment but decreased after enzymatic treatment. All *Lactobacilli* showed lactic dehydrogenase activity and production of D‐ and L‐lactic acid isomers on *Lactobacillus* CFSs, while only 1A‐TV and 35A‐TV released hydrogen peroxide and carried *helveticin* J and *acidocin* A bacteriocins. These results suggest that they can be employed as a new vaginal probiotic formulation and bio‐therapeutic preparation against urogenital infections. Further, in vivo studies are needed to evaluate human health benefits in clinical situations.

## Graphical abstract

The present study characterized a novel combination of *Lactobacillus gasseri* 1A‐TV, *L. fermentum* 18A‐TV, and *L. crispatus* 35A‐TV as live strains and as non‐live cell‐free supernatants showing antimicrobial activity against the most common multidrug‐resistant urogenital pathogens (*Streptococcus agalactiae*, *Escherichia coli*, KPC‐producing *Klebsiella pneumoniae*, *Staphylococcus aureus*, *Enterococcus faecium* VRE, *Enterococcus faecalis*, *Pseudomonas aeruginosa*, *P. mirabilis*, *P. vulgaris*, *Candida albicans*, *C. glabrata*). Our findings are promising for their use as a new valid vaginal probiotic and bio‐therapeutic formulation against multidrug‐resistant pathogens.

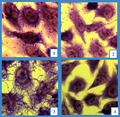

## 1 INTRODUCTION

*Lactobacilli* are important members of the human gastrointestinal, oral, and vaginal microbiota and are gaining great interest for their health‐promoting effects in the host both on direct interactions between cells and indirectly through their released metabolites, thus making them suitable to be used as probiotic strains (Reid et al., 2011). Over the last few years, the search for probiotic strains possessing innovative functional characteristics and formulations has been evolving and is an attractive goal in therapeutic strategies to restore the natural microbiota. Antibiotic treatment is the main approach used to fight bacterial infections (Aslam et al., 2018), but excessive and inappropriate use in both hospital and community settings has been one of the main factors of the onset of antibiotic resistance, and urogenital tract infections (UGTIs) are the most common infections in which many multidrug‐resistant (MDR) pathogenic strains are recorded due to the abuse of antibiotic therapy (Matulay et al., 2016).

*Lactobacilli* dominate the healthy vaginal microbiota and are considered gatekeepers of this ecosystem, maintaining a healthy state and impeding the growth of pathogens (Bautista et al., 2016; Martin, 2012; Ravel et al., 2011). Recent studies have focused on the vaginal microbiome in healthy reproductive‐aged women by 16S rRNA gene sequencing showed at least 5 community state types (CSTs), in which four were dominated by *L*. *crispatus* (CST‐I), *L*. *gasseri* (CST‐II), *L*. *iners* (CST‐III), *L*. *jensenii* (CST‐V), and only one by the microbial community (CST‐IV) composed of polymicrobial species confirming an important protection factor of the *Lactobacillus* population against potential pathogens associated with urogenital tract infections (UTIs) (Borges et al., 2014; Eryilmaz et al., 2018; Razzak et al., 2011; Wijgert et al., 2014). However, besides the most abundant vaginal *Lactobacilli*, other species have been encountered in the healthy vaginal microbiota such as *L*. *rhamnosus*, *L*. *fermentum*, *L*. *plantarum*, *L*. *brevis*, *L*. *casei*, *L*. *delbrueckii*, *L*. *vaginalis*, and *L*. *salivarius* (Dimitonova et al., 2008; Kiss et al., 2007; Pino et al., 2019; Smith & Ravel, 2017).

Perturbations of this highly regulated ecosystem occur during urogenital tract infections (UGTIs), as well as urinary tract infections (UTIs), bacterial vaginosis (BV), and during antimicrobial therapy, resulting in an even greater aberration of the microbiota and, eventually, in the extension of an infectious state (Donders et al., 2017; Eryilmaz et al., 2018; Matulay et al., 2016). Restoration of vaginal homeostasis, driven by *Lactobacilli*, may be accomplished through numerous mechanisms: (i) “competitive exclusion,” the first critical line of defense against local pathogens, which is the ability of bacteria to adhere to vaginal epithelial cells competing for nutrients and adhesion receptors (Liu et al., 2008), (ii) “co‐aggregation,” the assembly of microbial communities into distinct, interlinked structures (Pino et al., 2019); in addition, (iii) an intense production of antimicrobial compounds such as lactic acid, hydrogen peroxide (H_2_O_2_), bacteriocin‐like substances, and biosurfactants may inhibit pathogen growth (Petrova et al., 2015).

In vitro and in vivo studies have indicated the use of probiotics as an alternative approach for restoring healthy vaginal microbiota by interfering with potential pathogens. Although the use of live microorganisms is currently widely employed, safety issues remain a matter of debate, mainly for vulnerable subjects (Borges et al., 2014; Ravel et al., 2011; Reid et al., 2011). To overcome these issues, in the last decade, the use of non‐live microorganisms such as heat‐killed probiotics, microbial extracts, and cell‐free supernatants has been growing in interest for their applications in therapeutic strategies also considering that they can confer relevant beneficial effects (Piqué et al., 2019).

In this study, we characterized three vaginal *Lactobacilli*, *L*. *gasseri* 1A‐TV, *L*. *fermentum* 18A‐TV, and *L*. *crispatus* 35A‐TV from healthy vaginal microbiota for their probiotic properties mainly focusing on their antimicrobial activity against the most common MDR UGTI pathogens (Ahmed et al., 2019; Al‐Zahrani et al., 2019), considering also both adhesive properties and inhibitory substances released in their cell‐free supernatants (CFS).

## 2 METHODS

### 2.1 Sample collection and microbial growth conditions

*L*. *gasseri* 1A‐TV, *L*. *fermentum* 18A‐TV, and *L*. *crispatus* 35A‐TV were isolated from vaginal swabs taken from healthy premenopausal women without symptoms of vaginal or urinary tract infections during normal gynecological examinations for routine analyses at the Obstetrics and Gynecology Unit of the University Hospital of Catania, Italy. The authors received the strains for the subsequent analysis and their characterization. All *Lactobacillus* strains were grown on Man Rogosa and Sharpe (MRS) agar (Oxoid), incubated for 48 h at 37°C under anaerobic conditions, using the GasPakEZ Gas Generating Pouch Systems (BD). All *Lactobacilli* were taxonomically identified at the species level by amplification and sequencing of the *tuf* and 16S rRNA genes for accurate identification. Genomic DNA was extracted from overnight cultures of isolates in 5 ml of MRS and the *tuf* and16S rRNA genes were amplified. All PCR products obtained were purified using the QIAquick PCR gel extraction kit (Qiagen) and sequenced (Hütt et al., 2016; Marchisio et al., 2015; Ventura et al., 2003). Sequence analyses were performed using Gapped BLAST (Altschul et al., 1997).

The indicator strains were selected from our microbial bank at the MMAR*Lab* as having MDR profiles. The strains *Streptococcus* *agalactiae* GB022, *Enterococcus* *faecalis* EFS1, *Enterococcus* *faecium* 75 VRE (*van*A‐positive), *Staphylococcus* *aureus* (MSSA) SA3, *Pseudomonas* *aeruginosa* IF1, *Proteus* *vulgaris* IF3, *Proteus* *mirabilis* IF2, *Escherichia* *coli* GM1, *Klebsiella* *pneumoniae* 340 KPC (KPC‐3 positive), *Candida* *albicans* CA312, and *Candida glabrata* CG2824 were used as target microorganisms for the determination of antagonistic activity (Table 1). All clinical isolates had been tested for antimicrobial susceptibility profiles according to the interpretative standard of the European Committee on Antimicrobial Susceptibility Testing 2019 (EUCAST) (2019) and INTEGRAL SYSTEM YEASTS Plus (Liofilchem®) for antimycotic resistance profile.

**TABLE 1:** Clinical information and antimicrobial profiles of indicators strains used in this study

ID	Species	Infection disease	Source	Phenotypic resistance profile
GB022	*S*. *agalactiae*	asymptomatic	Vaginal swab	AK‐TOB‐LEV‐CIP‐LNZ‐TE‐TGC‐E‐DA‐RD
SA3	*S*. *aureus*	vaginitis	Vaginal swab	TOB‐AMC‐TZP‐LEV‐CIP‐E
EFS1	*E*. *faecalis*	vaginitis	Vaginal swab	FOS‐IPM‐TOB‐AMC‐LNZ‐F‐SXT‐CIP
75VRE	*E*. *faecium*	vaginitis	Vaginal swab	AMC‐TOB‐IPM‐TZP‐LEVCIP‐LNZ‐E‐QDA‐RD‐TEC‐VA
GM1	*E*. *coli*	Symptomatic cystistic	urine	AMC‐RD‐F
340KPC	*K*. *pneumoniae*	Symptomatic cystistic	urine	ETP‐MRP‐MEM‐AMC‐TZP‐C/T‐CAZ‐TGC‐RD‐F
IF1	*P*. *aeruginosa*	Symptomatic cystistic	urine	IPM‐MRP‐MEM‐TOB‐AMC‐TZP‐LEV‐CIP‐RD‐ATM‐SXT
IF2	*P*. *mirabilis*	Symptomatic cystistic	urine	FOS‐MRP‐MEM‐TOB‐AMC‐TZP‐C/T‐CAZ‐FEP‐CTZ‐LEV‐CIP‐TGC‐RD‐CS‐ATM‐SXT
IF3	*P*. *vulgaris*	Symptomatic cystistic	urine	ETP‐MRP‐MEM‐AMC‐C/T‐TGC‐RD‐CS
CA312	*C*. *albicans*	Vulvo‐vaginal candidosis	Vaginal swab	ECN‐KCA‐CLO‐MIC‐AMB*‐ITR*‐VOR*‐FLU*
CG2824	*C*. *glabrata*	Vulvo‐vaginal candidosis	Vaginal swab	CLO‐MIC‐ITR‐VOR‐FLU‐NY*‐AMB*‐ECN*‐KCA*

Abbreviations: AK, Amikacin; AMB, Amphotericin; AMC, Amoxicillin–clavulanic acid; ATM, Aztreonam; C/T, ceftolozane/tazobactam; CAZ, Ceftazidime; CIP, Ciprofloxacin; CLO, Clotrimazole; CN, Gentamicin; CS, Colistin; CTZ, Cefotaxime; CZA, Ceftazidime/avibactam; DA, Clindamycin; E, Erythromycin; ECN, Econazole; ETP, Ertapenem; F, Nitrofurantoin; FCY, Flucytosine; FEP, Cefepime; FLU, Fluconazole; FOS, Fosfomycin; IPM, Imipenem; ITR, Itraconazole; KCA, Ketoconazole; KPC, *Klebsiella* *pneumoniae* carbapenemase; LEV, Levofloxacin; LNZ, Linezolid; MIC, Miconazole; MRP, Meropenem; NY, Nystatin; QDA, Quinupristin–dalfopristin; RD, Rifampicin; SXT, Trimethoprim/Sulfamethoxazole; TE, Tetracycline; TEC, Teicoplanin; TGC, Tigecycline; TOB, Tobramycin; TZP, Piperacillin–tazobactam; VA, Vancomycin; VRE, vancomycin‐resistant enterococci; VOR, Voriconazole.

* intermediate resistance.

### 2.2 In vitro safety assessment of *Lactobacillus* strains

i. Antibiotic susceptibility testing and detection of hemolytic activity.

The antibiotic susceptibility profiles of the three *Lactobacilli* were determined by the Kirby‐Bauer diffusion and E‐test methods on MRS agar at 37°C for 48 h under anaerobic conditions (Charteris et al., 2007). The following antibiotics were tested: penicillin, ampicillin, amoxicillin–clavulanic acid, vancomycin, gentamicin, streptomycin, tetracycline, chloramphenicol, erythromycin, clindamycin, trimethoprim–sulfamethoxazole, rifampicin, ciprofloxacin, levofloxacin, and metronidazole. The antimicrobial susceptibility profiles were analyzed according to the interpretative standard of the European Union Commission recommendations for probiotic safety (Authority EFS, 2012).

ii. The hemolytic activity of *Lactobacilli* was visually verified on Columbia agar base supplemented with 5% sheep and horse blood (Oxoid) after 24 h and 48 h of incubation under anaerobic conditions at 37°C(Maragkoudakis et al., 2006). *Streptococcus pyogenes*, strain ATCC 19615, was used as a positive control. Both experiments mentioned above were performed in triplicate.

### 2.3 Determination of antagonistic activity

The MDR indicator strains, *S*. *agalactiae*, *E*. *faecalis* VRE, *E*. *faecium*, *S*. *aureus*, *P*. *aeruginosa*, *P*. *mirabilis*, *P*. *vulgaris*, *E*. *coli*, KPC‐producing *K*. *pneumoniae*, *C*. *albicans*, and *C*. *glabrata*, were used for detecting the antimicrobial activity of *Lactobacilli*. The inhibitory activity of vaginal strains was determined by the deferred antagonism test and quantified by the agar spot test with some modifications (Santagati et al., 2012; Siroli et al., 2017). In addition, for the evaluation of Lactobacillus combination, *L*. *gasseri* 1A‐TV, *L*. *fermentum* 18A‐TV, and *L*. *crispatus* 35A‐TV were grown in MRS broth for 48 h at 37°C under anaerobic conditions, using the GasPakEZ Gas Generating Pouch System (BD, New Jersey, USA) and approximately 2 × 108 CFU/ml of each *Lactobacillus* culture in a 1:1:1 ratio were used. Briefly, for the deferred antagonism assay, the test strain was inoculated diametrically across MRS agar with the addition of 0.1% CaCO_3_ and incubated for 48 h at 37°C under anaerobic conditions, as reported before. *Lactobacillus* growth was stopped, and the surface of the plate was sterilized by exposure to chloroform vapors for 30 min. The broth cultures of the indicator strains, grown for 18 h at 37°C, were streaked across the *Lactobacillus* growth line, and the plates were incubated for 18 h at 37°C to examine the interference zones with the indicator. *Lactobacillus* isolates that inhibited the growth of an indicator strain were considered inhibitory for that species (Maragkoudakis et al., 2006). For the agar spot test, the *Lactobacillus* cultures were spotted (5 µl) on the surface of MRS agar (1.2%) (20 ml) and incubated anaerobically for 48 h at 37°C. Then, 100 µl of an overnight culture of indicator strains (approximately 107 CFU/ml) was mixed with 10 ml of BHI soft agar (0.7%) and poured over the plate in which *Lactobacilli* were grown. After incubation for 24 h at 37°C, the inhibition zones around *Lactobacillus* spots were diametrically measured and expressed as diameter >10 (+ + + +); Diameter between 6 and 10 mm (+ + +); Diameter between 3 and 6 mm (+ +); Diameter between 1 and 3 mm (+); no inhibition (−) (Siroli et al., 2017).

### 2.4 Auto‐aggregation and co‐aggregation assays

Auto‐aggregation assays were performed according to Kos et al. (Kos et al., 2003). The auto‐aggregation percentage is expressed as A% = 1−(A_t5_/A_t0_) × 100, where A_t5_ represents the absorbance measured by a microplate reader (BioTek Synergy™ H1) at 600 nm after centrifugation at 650 × g for 2 min at time t = 5 h and A_t0_ the absorbance at t = 0. The percentage of co‐aggregation (CoA%) was calculated according to the equation of Malik et al. (Malik et al., 2003): CoA% = OD_TOT_−OD_S_/OD_TOT_ × 100, where the OD_TOT_ value represents total absorbance, taken immediately after the relevant strains were paired; and OD_S_ refers to the absorbance of the supernatant after 5 h from when the mixture was centrifuged. The statistical analysis was determined by ANOVA with Fisher's least significant difference (LSD) test, *p* < 0.05 (De Gregorio et al., 2014). Both tests were repeated in triplicate.

### 2.5 In vitro adhesion test

HeLa cells (ATCC^®^ CCL‐2.2 ^TM^) were grown in RPMI 1640 (Sigma‐Aldrich Inc, St. Louis, MO, USA) at 37°C with 5% CO_2_ supplemented with 10% (v/v) fetal bovine serum (FBS, Thermo Fisher Scientific), 1% (v/v) L‐glutamine, penicillin G (100 IU mL^−1^), and streptomycin (100 mg/L) (Sigma‐Aldrich). The *Lactobacillus* adhesion to the HeLa cell layer was performed on microscope cover glasses and expressed as percentage adherence. Briefly, *Lactobacillus* cultures grown anaerobically for 48 h at 37°C in MRS broth (Oxoid) were harvested by centrifugation (5000 × g for 15 min, 4°C), and the cells were washed twice with a sterile solution of 0.85% NaCl (w/v) (Sigma‐Aldrich) diluted in RPMI 1640 medium at 5 × 108 CFU/ ml and incubated with a monolayer of HeLa for 1 h at 37°C (Martín et al., 2020; Mastromarino et al., 2002). After washes, the cells were fixed with 3 ml of methanol and stained with 3 ml of Giemsa stain solution (1:20; Carlo Erba, Milan, Italy) for 30 min at room temperature. Wells were washed and dried at 30°C for 1 h. Adherent bacteria were examined microscopically by light‐microscopy DM5500 (Leica, Wetzlar, Germany) in 20 random microscopic fields to obtain *Lactobacillus* counts and averages. The adhesion indexes (ADI; the number of bacteria/100 HeLa cells) were expressed as strong adhesion: ADI >2500; good adhesion: good adhesion: ADI between 2500 and 500, weak adhesion between 500 and 100, no adhesion, ADI <100. Bacterial adhesion to the HeLa cell layer was also evaluated by viable counts. After incubation, supernatants were discarded and non‐adherent bacteria were removed by washing each well twice with PBS and after the detachment by 1 ml of PBS with 0.1% Triton X‐100 (Sigma‐Aldrich, USA). The viable counts of adherent lactobacilli were evaluated by CFU/ml on MRS agar plates after incubation anaerobically for 48 h at 37°C (Santagati et al., 2012).

### 2.6 Biofilm formation assay

Biofilm production was tested in MRS broth. *Lactobacillus* biofilm development was evaluated as described by Ibarreche et al. (Perez Ibarreche et al., 2014) with modifications. Briefly, 200 μl of the medium was added to each well of sterile 96‐well plates (Corning® Incorporated Life Sciences, NY, USA) and was inoculated with LAB cultures at 3 × 108 CFU/ml. The plates were incubated under anaerobiosis at 37°C for 72 h. To quantify the biofilm formation, the wells were washed 3 times with PBS, fixed for 1 h at 37°C, and then stained for 30 min with 200 μl of 2% (v/v) crystal violet. The excess dye was rinsed with sterile distilled water, and the plates were allowed to dry at room temperature. The dye that had adhered to the cells was resolubilized with 200 μl of 95% (v/v) ethanol, and the absorbance of each well was measured at 570 nm using a microplate reader (BioTek Synergy™ H1). We used *L*. *rhamnosus* GGATCC 53103 as a positive control strain as it was a good biofilm producer (Lebeer et al., 2007), and MRS medium without inoculum was included as a negative control. As a selection criterion for biofilm formation, a cutoff OD (ODc) for the test was defined as three standard deviations above the mean OD of the negative control. The strains were considered non‐biofilm producers (OD_ODc); weak biofilm producers (ODc<OD_2_ODc); moderate biofilm producers (2_ODc<OD_4_ODc); strong biofilm producers (4_ODc<OD_8_ODc); and very strong biofilm producers (8_ODc<OD). These experiments were performed in triplicate.

### 2.7 Assessment of in vitro antimicrobial activity of *Lactobacillus* cell‐free supernatants

Cell‐free supernatants (CFSs) of *L*. *gasseri* 1A‐TV, *L*. *fermentum* 18A‐TV, and *L*. *crispatus* 35A‐TV and the CFS of the *Lactobacilli* combination were prepared as previously reported (Parolin et al., 2015). Each *Lactobacillus* culture was centrifuged at 7000 × g for 30 min at 4°C, and their supernatants were filtered through a 0.2 μm membrane, and pH values were measured by a pH meter (pH50+DHS Bench pH meter). For the CFS combination, each *Lactobacillus* culture at 2 × 108 CFU/ml, after the filtration step, was mixed in a 1: 1:1 ratio.

The antimicrobial activity of CFSs was assayed against the indicator strains previously mentioned. The antagonism experiment was performed in a sterile 96‐well plate (Corning^®^ Incorporated Life Sciences, NY, USA) using the indicator strains at 3 × 105 CFU/ml. In each plate, 50 μl of *Lactobacillus* CFS was mixed with 50 μl of each indicator of antagonist tests and of control growth, 50 μl of sterile MRS medium and 50 μl of each indicator strain were mixed. The 96‐well plates were incubated at 37°C under aerobic conditions and evaluated at 6 h and 24 h.

The results were considered by evaluating the growth inhibition of the indicator strains. The viable microbial cell counts (CFU/ml) of each indicator strain were recorded as log_10_ reduction of the total count of CFU/ml in the original inoculum, planting on Mueller–Hinton agar (Oxoid, Basingstoke, UK) and Sabouraud dextrose agar plates (BD). The bactericidal activity was defined as a reduction of at least 99.9% (≥3 log_10_) (NCCLS, 1999). This experiment was repeated in triplicate.

### 2.8 Evaluation of the antimicrobial activity of CFSs after pH, heat, catalase, and proteolytic enzymatic treatment

The effects of heat treatment, catalase, and proteolytic enzymatic treatments were evaluated for all CFSs. The effect of temperature was determined by exposing 5 ml of each aliquot of CFS to 70°C and 100°C for 30 min and 121°C for 15 min. The sensitivity of the CFSs to enzymatic activity was assayed by catalase (E. C.1.11.1.6) at pH 7.0 (50 mM potassium phosphate buffer), trypsin (E. C.3.4.21.4, type II), and proteinase K (E. C. 3.4.21.64) at pH 7.5 (100 mM Tris‐HCl buffer). Aliquots of the CFSs were incubated (1:1 v/v) with enzyme solutions (1 mg/ml) and their respective controls at 37°C for 2 h under aerobic conditions (Oliveira et al., 2017). After these treatments, the antibacterial activity of the CFSs was determined by antagonism experiments in 96‐well plates and expressed as total (+++), good (++), partial (+), and no inhibition (‐). The effects of pH were tested at pH 5.5, 6.5, and 7.5 adjusted by 10 N NaOH, and untreated cell‐free supernatants were used as controls. The antagonism experiments were performed in a sterile 96‐well plate (Corning^®^ Incorporated Life Sciences) using the indicator strains at 3 × 105 CFU/ml as described above. After incubation for 6 and 24 h at 37°C, the results were estimated by the growth rates of the indicator strains measured by a turbidimetric method with Microplate Reader (BioTek Synergy™ H1) system using OD_600_ for bacterial strains and OD_530_ for *Candida* spp. (Yang et al., 2018). All experiments were repeated three times.

### 2.9 Determination of hydrogen peroxide production, lactic dehydrogenase activity, L‐ and D‐lactic acid production, and the presence of bacteriocin genes

The production of H_2_O_2_ was tested by the Eschenbach method (Eschenbach et al., 1989) using the scale previously reported by Parolin et al. (Parolin et al., 2015). All strains were scored as low (score 1 [>20 min]), medium (score 2 [10–20 min]), or high (score 3 [<10 min]) producing strains. Isolates not producing blue coloration were scored as 0. We used *L*. *acidophilus* ATCC 4356 as a positive control strain for H_2_O_2_ production. To determine the activity of lactic dehydrogenase (LDH), the cells were harvested after 48 h at 37°C under anaerobic conditions at an optical density (OD_600 nm_) of 1.5 and centrifuged at 10000 × g for 10 s. The cells were washed and resuspended in 2 ml of phosphate‐buffered saline (PBS; 137 mmol/L NaCl, 2.7 mM KCl, 10 mM Na_2_HPO_4_·12H_2_O, 1.8 mM KH_2_PO_4_, pH 7.4). The cell suspensions were ultrasonicated using a BANDELIN SONOPULS HD 2070 sonicator. The LDH activity of bacterial cell lysates from 1A‐TV, 18A‐TV, and 35A‐TV strains was determined through the kinetics of the decrease in NADH absorbance (Δmin) that was measured by a spectrophotometer (Hitachi U‐2000) at *λ* = 340 nm (Kasai et al., 2019). The enzyme assay was performed at 30°C, and 1 U of the enzyme was defined as the amount of enzyme that catalyzes the degradation of 1 µmol of NADH per minute (Sung et al., 2004).

The production of D‐ and L‐lactic acid produced by *Lactobacillus* were determined on cell‐free supernatants using a commercial assay kit (Cat. No.11112821035, R‐Biopharm) according to the manufacturer's instructions, the kit used the internal control solutions for the enzymatic determination. The lactic acid production was expressed in g/L. In both tests, lactic dehydrogenase activity, L‐ and D‐lactic acid production, we used *L*. *rhamnosus* *GG*ATCC 53103, lactic acid producer as a control strain. The probiotic *L*. *rhamnosus* *GG*ATCC 53103 had lactic dehydrogenase activity (46 U mg/L) and was L‐lactic acid (2.8 g/L) and D‐lactic acid (0.03 g/L) producer (Allonsius et al., 2019). The detection of bacteriocin‐encoding genes was conducted by analyzing those most frequently present in the *Lactobacilli* species: *nisin*A, *nisin*B, *nisin*F, *gassericin*A, *gassericin*T, *gassericin*K, *gassericin*E, *lactacin*F, *helveticin*J, *acidocin*A, *acidocin*B, *plantericin*A, *plantericin*EF, and *pediocin*A. The primers used, designed by Vector NTI software, are listed in Table A1. PCR was performed as previously published (Santagati et al., 2012).

### 2.10 Statistical analysis

Statistical analyses were performed using GraphPad Prism 6 software (GraphPad Software Inc.), and results were expressed as mean ±standard deviation (SD) of 3 independent experiments. For the co‐aggregation assays, ANOVA with Fisher's least significant difference (LSD) test was used to determine significant differences (*p* < 0.05).

## 3 RESULTS

### 3.1 Evaluation of *Lactobacillus* antagonistic activity against multidrug‐resistant clinical isolates

The antagonistic activity of *L*. *gasseri* 1A‐TV, *L*. *fermentum* 18A‐TV, and *L*. *crispatus* 35A‐TV, assessed by the agar spot test, showed the best growth inhibition with diameters >10 mm (+ + + +) for *E*. *coli* GM1, *S*. *aureus* SA3, *E*. *faecalis* EFS1, *S*. *agalactiae* GB022, *E*. *faecium* 75 VRE and for *K*. *pneumoniae* 340 KPC, multidrug‐resistant pathogens frequently associated with serious infections. All three *Lactobacilli* antagonized *C*. *albicans*, showing inhibition zones between 6 and 10 mm (+ + +), and exerted a partial inhibition versus *C*. *glabrata*, with inhibition zones between 1 and 3 mm. They also showed good inhibition versus *P*. *aeruginosa*, *P*. *mirabilis*, and *P*. *vulgaris* with diameters between 3 and 6 mm (++). The same results were obtained with the combination (1:1:1 ratio) of the three *Lactobacilli* (Table 2).

**TABLE 2:** In vitro inhibitory activity against UGTI pathogens (indicator strains), H_2_O_2_production, bacteriocin gene, lactic dehydrogenase activity, detection and sensitivity of the CFS antimicrobial activity to heat, catalase, and proteolytic enzymatic treatment of vaginal *Lactobacilli* isolates 1A‐TV, 18A‐TV, 35 A‐TV, and their combination

	Bacteriocingenes	Lactic dehydrogenase activity	H_2_O_2_production test^b^	Indicators strains			*S. agalactiae* GB022	*E. coli* GM1	*K. pneumonia* 340KPC	*S. aureus* SA3	*E. faecium* 75VRE *E. faecalis* EFS1	*P. aeruginosa* IF1	*P. vulgaris* IF1 *P. mirabilis* IF3	*C. albicans* CA312	*C. glabrata* CG2824
1 A‐TV*L. gasseri*	Acidocin A, Helveticin J	25.57 U mg/1	High	Deferred agar spot assay^a^			*+ + + +*	*+ + + +*	*+ + + +*	*+ + + +*	*+ + + +*	*++*	*++*	*+ + +*	*+*
				Untreated‐pH4.2			+++	+++	+++	+++	+	+++	+++	−	−
					Enzymes^c^	Trypsin	‐	+	+	−	−	+++	−	−	−
						Proteinase K	+	+	+	+	−	+++	−	−	−
						Catalase	+	+	+	+	−	++	−	−	−
					Temperature^c^	70°C, 30 min	+++	+++	+++	+++	+	+++	+++	−	−
						100°C, 30 min	+++	+++	+++	+++	+	+++	+++	−	−
						121°C, 15 min	+++	+++	+++	+++	+	+++	+++	−	−
18 A‐TV *L. fermentum*	No bacteriocin	28.27 U mg/1	0	Deferred agar spot assay^a^			*+ + + +*	*+ + + +*	*+ + + +*	*+ + + +*	*+ + + +*	*++*	*++*	*+ + +*	*+*
				Untreated‐pH4.3			+++	++	+++	+++	+	+++	+++	−	−
					Enzymes^c^	Trypsin	−	+	++	−	−	+++	+++	−	−
						Proteinase K	−	+	−	−	−	+++	+++	−	−
						Catalase	+++	++	+++	+++	+	+++	+++	−	−
					Temperature^c^	70°C, 30 min	+++	++	+++	+++	+	+++	+++	−	−
						100°C, 30 min	+++	++	+++	+++	+	+++	+++	−	−
						121°C, 15 min	+++	++	+++	+++	+	+++	+++	−	−
35 A‐TV*L. crispatus*	Acidocin A	56.17 U mg/1	Low	Deferred agar spot assay^a^			*+ + + +*	*+ + + +*	*+ + + +*	*+ + + +*	*+ + + +*	*++*	*++*	*+ + +*	*+*
				Untreated‐pH4.8			+++	+++	+++	+++	+	+++	+++	−	−
					Enzymes^c^	Trypsin	−	−	−	−	−	−	−	−	−
						Proteinase K	−	−	−	−	−	+++	−	−	−
						Catalase	−	−	−	−	−	++	−	−	−
					Temperature^c^	70°C, 30 min	+++	++	++	++	+	+++	+++	−	−
						100°C, 30 min	+++	++	++	++	+	+++	+++	−	−
						121°C, 15 min	+++	++	++	++	+	+++	+++	−	−
Lactobacilli Mix	Acidocin A, Helveticin J	N.D	N.D	Deferred agar spot assay^a^			*+ + + +*	*+ + + +*	*+ + + +*	*+ + + +*	*+ + + +*	*++*	*++*	*+ + +*	*+*
				Untreated‐pH4.4			+++	+++	+++	+++	+	+++	+++	−	−

^a^ Interpretation criteria for the deferred agar spot test: Diameter > 10 (+ + + +); Diameter between 6 and 10 mm ( + + +); Diameter between 3 and 6 mm (+ +); Diameter between 1 and 3 mm (+); no inhibition (−).

^b^ Interpretation criteria for H_2_O_2_ production: low (score 1[>20 min]), medium (score 2[10–20 min]); high (score 3 [<10 min]), no production 0, ND: undefined.

^c^ Interpretation criteria for antagonistic activity after, heat, catalase, and proteolytic enzymatic treatment: total (+++), good (++), partial (+), and no inhibition (***−***).

### 3.2 In vitro safety assessment

*L*. *gasseri* 1A‐TV, *L*. *fermentum* 18A‐TV, and *L*. *crispatus* 35A‐TV were sensitive to penicillin G, ampicillin, amoxicillin–clavulanic acid, tetracycline, chloramphenicol, erythromycin, rifampicin, and clindamycin. Intrinsic resistance to trimethoprim–sulfamethoxazole, metronidazole, gentamicin, levofloxacin, ciprofloxacin, streptomycin, and vancomycin was confirmed, except for *L*. *gasseri* that was sensitive to vancomycin. Safety assessment tests showed that none of the tested *Lactobacilli* caused the complete lysis (*β*‐hemolysis) of erythrocytes on sheep and horse blood agar. The in vitro safety assessment of vaginal *Lactobacilli* isolates is given in Table A2.

### 3.3 Aggregation assays and biofilm formation

Aggregation properties were assayed with the auto‐aggregation and co‐aggregation tests measuring two characteristics of the strains. Auto‐aggregation can be mediated by intra‐species cellular promoting factors and cell‐wall hydrophobicity, while co‐aggregation is the ability to achieve an adequate mass by co‐aggregating other bacterial species, however, the ability of a probiotic to aggregate is a desirable property.

The auto‐aggregation rates of *L*. *gasseri* 1A‐TV, *L*. *fermentum* 18A‐TV, and *L*. *crispatus* 35A‐TV, measured after 5 h of incubation, gave the following values: 75.14% ±0.01, 79.41% ±0.01, 83.10% ±0.02, respectively. The degree of *Lactobacilli* co‐aggregation with *S*. *agalactiae*, *E*. *coli*. *K*. *pneumoniae*, *S*. *aureus*, *E*. *faecium*, *E*. *faecalis*, *P*. *aeruginosa*, *P*. *vulgaris*, and *P*. *mirabilis* was very high, ranging between 51.3 ± 0.02 and 83.19 ± 0.03. *C*. *albicans* and *C*. *glabrata*, despite a strong value of selective interactions versus *Lactobacilli* strains (83.51, 89.58%, respectively), possessed a strong auto‐aggregation property (95.1 and 96.8%, respectively).

**FIGURE 1:**
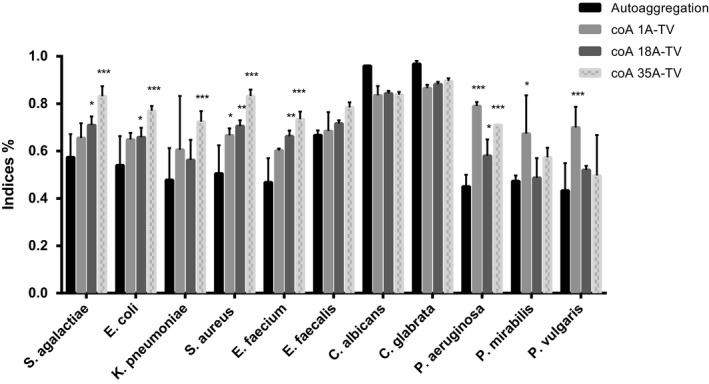
Co‐aggregation ability of *Lactobacilli* after 5 h incubation at room temperature in PBS (pH 7.4). Results are presented as the average of at least three independent experiments, and the error bars correspond to standard deviations. Statistical significance was evaluated by ANOVA with Fisher’s least significant difference (LSD) (*p ≤ 0.05, **p ≤ 0.01, ***p ≤ 0.001)

Despite the co‐aggregation percentage of all bacterial strains being higher than self‐aggregation percentages, significant co‐aggregation (*p* < 0.05) was found only for *S*. *aureus*, *P*. *aeruginosa*, and *Proteus* spp. with 1A‐TV, *S*. *agalactiae*, *E*.*coli*, *S*. *aureus*, *E*. *faecium*, and *P*. *aeruginosa* with 18 A‐TV and 35A‐TV; in addition, 35A‐TV also showed significant co‐aggregation with *K*. *pneumoniae* (Figure 1). Regarding the biofilm production, we found different levels: weak for *L*. *gasseri* 1A‐TV, moderate for *L*. *fermentum* 18A‐TV while *L*. *crispatus* 35A‐TV was not a biofilm producer; however, the *Lactobacillus* combination stood out as being a strong biofilm producer.

### 3.4 Adhesion test on HeLa cells

**FIGURE 2:**
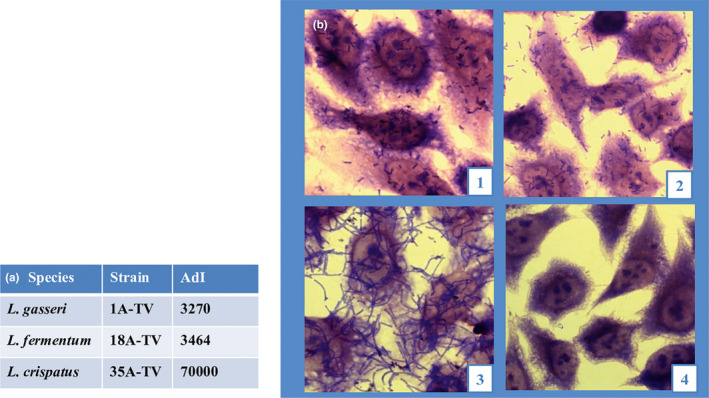
Bacterial adhesion to HeLa cell layer. (a) Adhesion indexes (ADI; the number of bacteria/100 HeLa cells). (b) Cell layers observed after Giemsa staining using light microscopy: (1) *L. gasseri* 1A‐TV; (2) *L. fermentum* 18A‐TV; (3) *L. crispatus* 35A‐TV; (4) Adhesion to HeLa cell monolayer as a negative control

*L*. *gasseri* 1A‐TV, *L*. *fermentum* 18A‐TV, and *L*. *crispatus* 35A‐TV were tested for their capability to adhere to HeLa cells. After being extensively washed with PBS, a significant proportion of cells from all bacterial strains remained attached to the HeLa monolayer displaying a strong adhesive phenotype, coinciding with an adhesion index (ADI) greater than 2500, as shown in Figure 2 (a, b). This adhesion in *L*. *crispatus* 35A‐TV showed an extraordinary ADI of 70000. The *Lactobacillus* adhesion was also tested by viable counts showing that *L*. *crispatus* 35A‐TV (6x10^6^± 0.24 CFU/ml) *L*. *gasseri* 1A‐TV (4,5×10^5^± 0.47 CFU/ml) and *L*. *fermentum* 18A‐TV (2,8810^5^± 0.38 CFU/ml) displayed a good ability to adhere to HeLa cells.

### 3.5 In vitro antimicrobial activity of *Lactobacilli* CFSs and their sensitivity to pH, heat, catalase, and proteolytic enzymatic treatment

**FIGURE 3:**
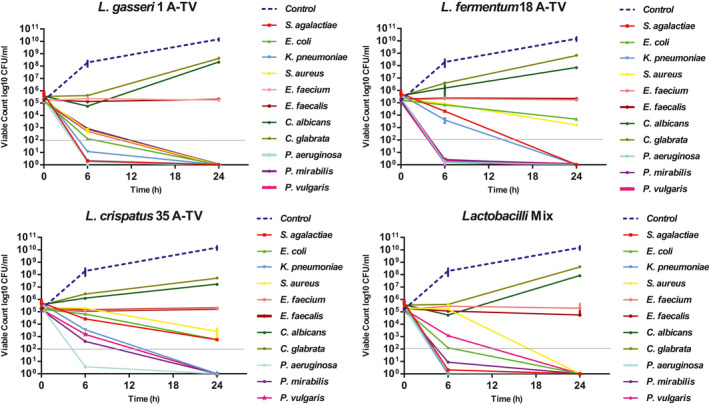
In vitro antimicrobial activity of cell‐free supernatants (CFSs) on indicator strains by time‐killing curves analysis. The gray dotted line indicates a 3‐log_10_ decrease in the number of CFU/ml versus the number at the baseline (bactericidal effect), while the blue dotted line indicates a general control growth

**FIGURE A1:**
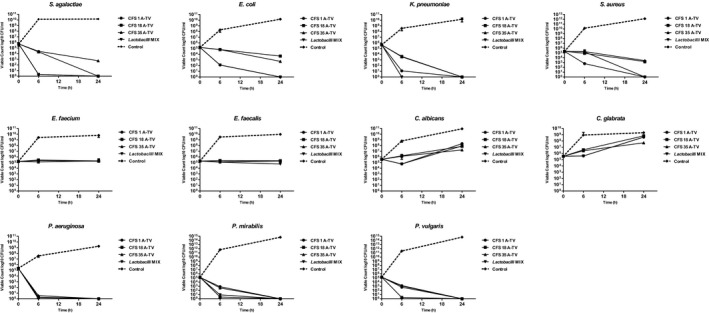
In vitro antimicrobial activity of cell‐free supernatants (CFSs) on each single indicator strain by time‐killing curve analysis. *The black dotted line indicates a growth control of each indicator strain, the solid black line with circles indicates the CFS of *L. gasseri* 1A‐TV, while the line with rectangles indicates the CFS of *L. fermentum* 18A‐TV, the line with triangles indicates the CFS of *L. crispatus* 35 A‐TV, and the line with triangles facing down indicates the CFS *Lactobacilli* mix

Cell‐free supernatants of *L*. *gasseri* 1A‐TV, *L*. *fermentum* 18A‐TV, and *L*. *crispatus* 35A‐TV, at pH 4.2, 4.3, and 4.8, respectively, were assayed for their ability to inhibit the pathogens by time‐killing tests (Figure 3 and Figure A1). After 6 and 24 h of incubation, *L*. *gasseri* 1A‐TV CFS could inhibit the growth of *S*. *agalactiae*, *E*. *coli*, *K*. *pneumoniae*, *S*. *aureus*, *P*. *aeruginosa*, *P*. *mirabilis*, and *P*. *vulgaris* exhibiting bactericidal activity. *L*. *fermentum* 18A‐TV reduced the growth by 1 log_10_ for *S*. *agalactiae*, *E*. *coli*, and *S*. *aureus* at T_6 h_, while it reduced the growth by 2 log_10_ for *K*. *pneumoniae*. At T_24 h,_ 18A‐TV reduced the growth by 2 log_10_ for *E*. *coli*, whereas it exhibited a complete inhibition (bactericidal effect) of *S*. *agalactiae*, *S*. *aureus*, and *K*. *pneumoniae*. Furthermore, *L*. *fermentum* 18A‐TV had bactericidal activity at both T_6 h_ and T_24 h_ against *P*. *aeruginosa*, *P*. *mirabilis*, and *P*. *vulgaris*.

Regarding *L*. *crispatus* 35A‐TV, no effect was found for *S*. *agalactiae*, *E*. *coli*, *K*. *pneumoniae*, *S*. *aureus*, and *P*. *vulgaris* at T_6 h_, but switched to bactericidal at T_24 h_, for *K*. *pneumoniae* and *P*. *vulgaris*, whereas 35A‐TV had a bactericidal activity from T_6 h_ against *P*. *aeruginosa* and *P*. *mirabilis*.

Moreover, all three *Lactobacilli* CFSs showed no antimicrobial effect versus *E*. *faecium* and *E*. *faecalis* at T_6 h_ and T_24 h,_ as well as both strains of *Candida* spp. In addition, the curve of candidal growth maintained a concentration similar to the initial inoculum (10^5^–10^6^ CFU/ml) also at T_24 h_ compared to the CFS‐free curve that reached a concentration of 10^9^–10^10^ CFU/ml.

Also, the inhibitory effect of the CFS combination (the three *Lactobacilli* together), evaluated at 6 h and 24 h, showed bactericidal activity only against *S*. *agalactiae*, *E*. *coli*, *K*. *pneumoniae*, *S*. *aureus*, *P*. *aeruginosa*, *P*. *vulgaris*, and *P*. *mirabilis*.

In Table 2, the effects of heat treatment and proteolytic enzymes on the CFS activity of each strain and their combination are shown. The treatment at 70, 100, and 121°C of the CFSs of *L*. *gasseri* 1A‐TV and *L*. *fermentum* 18A‐TV did not alter their antagonistic activity, whereas a reduction was observed only for *L*. *crispatus* 35A‐TV CFS versus *E*. *coli*, *K*. *pneumoniae*, and *S*. *aureus*. The catalase treatment decreased inhibitory activity for 1A‐TV and 35A‐TV CFSs, while no effects were observed in 18A‐TV.

Regarding proteolytic treatment, we registered the loss, or the strong reduction, of the inhibitory activity of the three singular supernatants versus *S*. *agalactiae*, *E*. *coli*, *S*. *aureus*, *K*. *pneumoniae*, *E*. *faecalis*, and *E*. *faecium*. The growth of *Proteus* spp. was inhibited by 18A‐TV CFS and maintained with 1A‐TV and 35A‐TV CFSs. Only for *P*. *aeruginosa*, was the bactericidal effect maintained by the three *Lactobacilli* CFSs after proteolytic treatment, except for *L*. *crispatus* 35A‐TV CFS after trypsin treatment.

**FIGURE A2:**
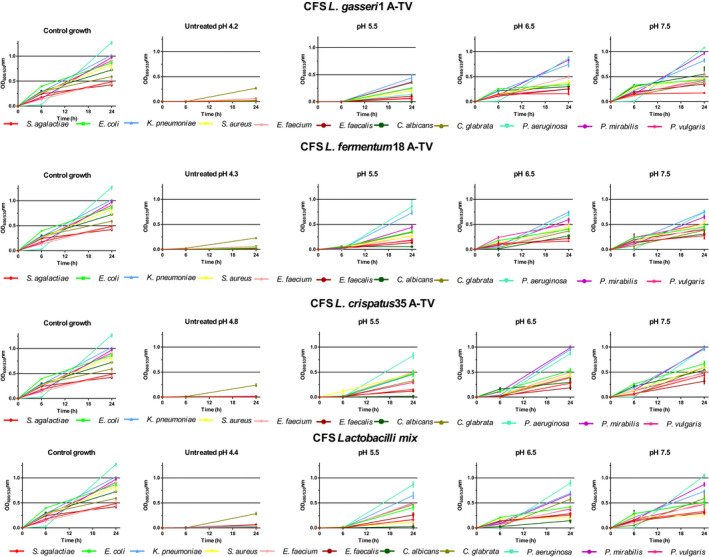
Effects of pH on CFS antimicrobial activity on indicator strain growth. Comparison of growth curves at 6 and 24h (OD = 600 nm for bacterial strains and OD = 530 for *Candida* spp.) of control indicator strains with respect to the growth of untreated *Lactobacilli* CFS, CFS adjusted to pH 5.5, 6.5, and 7.5. The results are expressed as mean ± standard deviations of values obtained from triplicate experiments

The pH‐dependent effects on antimicrobial activity of CFSs were tested at pH 5.5, 6.5, and 7.5. by measurements of the growth rates (OD) of the indicator strains. All CFS *Lactobacilli* and their formulation at pH 5.5 maintained their activity up to 6 h and weakly lost their efficiency at 24 h compared to the untreated pH, while the antagonistic activity of CFS at pH 6.5 and 7.5 was lost after 6 h despite the growth of the indicator curve showed a slight decrease in slope compared to controls (Figure A2).

### 3.6 Determination of hydrogen peroxide production, lactic dehydrogenase activity, and bacteriocin‐encoding genes

*L*. *gasseri* 1A‐TV produced a higher quantity of hydrogen peroxide with respect to *L*. *crispatus* 35A‐TV, while *L*. *fermentum* 18A‐TV did not release this metabolite (Table 2).

The lactic dehydrogenase activity was evaluated using cell lysates, in particular, *L*. *crispatus* 35A‐TV had a specific activity of 56.17 U mg/L, higher than that observed in *L*. *gasseri* 1A‐TV and *L*. *fermentum* 18A‐TV, which were, respectively, 25.57 U mg/L and 28.27 U mg/L (Table 2). In addition, all *Lactobacilli* were L‐ and D‐lactic acid producers showing the production of D‐lactic acid 4.04 (g/L), 3.7 (g/L), 3.11 (g/L), and L‐lactic acid between 2.94 (g/L), 2.95 (g/L), and 3.34 (g/L) for *L*. *gasseri* 1A‐TV, *L*. *fermentum* 18A‐TV, and *L*. *crispatus* 35A‐TV, respectively.

The detection of bacteriocin‐encoding genes revealed helveticin J only in *L*. *gasseri* and acidocin A in *L*. *gasseri* and *L*. *crispatus*. *L*. *fermentum*, despite showing activity against pathogens, was negative for all genes tested (Table 2).

## 4 DISCUSSION

Several studies have reported beneficial effects exerted by probiotics, and it has been well demonstrated that functional properties are strain‐dependent (Borges et al., 2014). In this study, we characterized three *Lactobacilli*, *L*. *gasseri* 1A‐TV, *L*. *fermentum* 18A‐TV, and *L*. *crispatus* 35A‐TV isolated from the vaginal microbiota, with the activities of their metabolites produced by CFSs for their beneficial features addressed mainly to their antimicrobial activity against multidrug‐resistant clinical isolates.

In accordance with the objectives of our study, the selected *Lactobacilli* were tested in vitro for surface properties to determine their capability to colonize the human vagina. In vitro experiments showed their ability to adhere to HeLa cells, and this is also related to their predisposition to self‐aggregate. As is well known, adhesion and auto‐aggregation represent the determining factors for the initial development of biofilm, which is a strategy of some organisms to persist in harsh environments promoting microbial resistance to antimicrobial agents, the immune system, and stress conditions (Leccese Terraf et al., 2016). In this regard, our *Lactobacilli* possessed strong biofilm formation capacity when tested in combination; however, they were poor producers when tested alone. These data make us hypothesize a synergistic interspecific interaction between our *Lactobacilli* to optimize their living conditions. Biofilm formation is a phenomenon that can promote mucosal colonization and masking epithelial cell receptors, can exert a protective role by interfering with the growth and adhesion of pathogens (Leccese Terraf et al., 2016).

Another mechanism that promotes an exclusion/competition behavior is the ability of beneficial bacteria to co‐aggregate with pathogens (Santos et al., 2016). In this regard, our *Lactobacillus* strains showed a significant capability to co‐aggregate with *S*. *agalactiae*, *E*. *coli*. KPC‐producing *K*. *pneumoniae*, *S*. *aureus* *E*. *faecium* VRE and *E*. *faecalis*, *P*. *aeruginosa*, *P* *vulgaris*, and *P*. *mirabilis*. This is an important contributing factor to create a microenvironment where pathogens can be exposed to higher concentrations of inhibitory substances or metabolites such as organic acids (e.g., lactic acid) and hydrogen peroxide mainly produced by *Lactobacilli* strains as the dominant bacterial population in the vaginal ecosystem (Verdenelli et al., 2014).

In this study, we found that cell‐free supernatants released from three *Lactobacilli* as single entities, and their combination, exhibited an antagonistic effect against multidrug‐resistant clinical isolates including *S*. *agalactiae*, *E*. *coli*, KPC‐producing *K*. *pneumoniae*, S. *aureus*, *E*. *faecium* VRE, *E*. *faecalis*, *P*. *aeruginosa*, *P*. *mirabilis*, and *P*. *vulgaris*.

Conversely, the anti‐candida activity of the three *Lactobacilli* showed different behavior with the two approaches: agar diffusion and using cell‐free supernatants, which had no growth‐inhibitory activity and could maintain the candidal growth almost at the same concentration as the initial inoculum compared to the control (CFS‐free). These conflicting results could be explained by the physical state of the media; the concentration of antimicrobial substances released into the solid and liquid media, and by the environment where the substances exert their effects. *Scorzoni* et al. also reported that the microdilution test is more sensitive to agar diffusion in the evaluation of anti‐candida activity highlighting the need to apply different methods to evaluate in vitro antimicrobial effects of *Lactobacilli* (Scorzoni et al., 2007).

Notably, the CFS combination maintained the same antagonistic profile of each strain, excluding a possible interference between them.

The activity of *Lactobacillus* CFSs after the heat and enzymatic treatments was reduced in some cases compared with untreated CFSs hypothesizing the presence of thermostable and thermosensitive substances such as bacteriocins in the supernatants, while the neutralization treatment at pH 6.5 and 7.5 canceled antagonistic effects. These data suggested that the acid environment and antimicrobial metabolites released by our strains such as bacteriocins had synergetic action against the growth of pathogens tested, showing a better antagonistic activity. Further, several reports suggested that the pH‐induced alterations of net charge might facilitate the translocation of some bacteriocin molecules through the cell wall (Oliveira et al., 2017) and that an acid environment could interfere with the production and bactericidal activity of several bacteriocins (Yang et al., 2018). pH and lactic acid levels display a strong inverse correlation demonstrating that lactic acid is the main acidifier of the human vagina, increasing its production under hypoxic conditions (Tachedjian et al., 2017), which displays antimicrobial and anti‐inflammatory properties. In this context, all three isolated *Lactobacilli* can produce D‐ and L‐lactic acid that could mainly contribute to the vaginal health promotion having also anti‐inflammatory effects (Alvarez‐Olmos et al., 2004; José Aníbal Mora‐Villalobos JM‐Z, 2020). Moreover, *Lactobacillus* production of hydrogen peroxide as diffusible inhibitory substances could be connected to antimicrobial properties of the vaginal microbiota, representing an important nonspecific antimicrobial defense mechanism due to a highly toxic state (Kullisaar et al., 2002; Mijac et al., 2006).

However, hydrogen peroxide production by *Lactobacilli* as well as *L*. *gasseri* 1A‐TV and *L*. *crispatus* 35A‐TV can be considered an additional beneficial effect for vaginal health (Antonio et al., 2005; Pendharkar et al., 2013), in vivo, conversely, under microaerobic (hypoxic) conditions such as the cervicovaginal environment, the concentration of H_2_O_2_ produced does not achieve the amount necessary to have antimicrobial activity in the vaginal environment. The low vaginal O_2_ levels measured in in vivo studies have been associated with little or no H_2_O_2_ in the hypoxic cervicovaginal environment (O'Hanlon et al., 2011). Therefore, these findings support an important role of lactic acids as main products at high concentrations in a hypoxic environment such as the vagina. However, H_2_O_2_ production remains an in vitro marker for beneficial vaginal properties (Tachedjian et al., 2018).

Additionally, bacteriocins are believed to contribute to the competitiveness between strains by acting against pathogenic strains; therefore, the production of bacteriocins represents an important antimicrobial factor (Soltani et al., 2020). Among our strains, *L*. *gasseri* 1A‐TV and *L*. *crispatus* 35A‐TV are producers of helveticin J and acidocin A, which is a small thermostable peptide with maximum production at pH 5, exerting antagonistic activity versus several bacterial genera, including *Lactococcus*, *Pediococcus* *Staphylococcus*, *Enterococcus*, *Streptococcus*, *Listeria*, *Clostridium*, and *Bacillus* (Kanatani et al., 1995). The stability at a high temperature of acidocin A and its bacterial targets suggested its decisive role in the antimicrobial activity exerted by the supernatants of *L*. *gasseri* 1A‐TV and *L*. *crispatus* 35A‐TV against the indicators tested, also considering that *L*. *crispatus* is considered a major determinant in the stability of the normal vaginal microbiota in women of reproductive age (Miller et al., 2016)

Our study strengthens the concept of using probiotic *Lactobacillus* to protect the host against MDR pathogens including *E*. *faecium* VRE and KPC‐producing *Klebsiella* *pneumoniae*, based on the antimicrobial activity of our *Lactobacilli*, *L*. *gasseri* 1A‐TV, *L*. *fermentum* 18A‐TV, and *L*. *crispatus* 35A‐TV and their combination, as well as their CFSs, showed clear antibacterial activity against multidrug‐resistant pathogens. Moreover, the three *Lactobacilli*, with some intra‐species diversity, share many probiotic features both as live and non‐live bacteria such as their released metabolites (CFSs) possessing the potential of colonizing the vaginal epithelium, producing antagonistic metabolites, and keeping their activity in different environmental conditions. Taken together, all these results support novel therapeutic strategies as a new vaginal formulation for the prevention and treatment of urogenital infections, acting on the rebalance of the vaginal microbiome.

Further experiments are planned to complete the characterization of these CFSs, with a more detailed knowledge of their metabolic profiles to better understand their nature and mode of action. Future work will also characterize the probiotic potential of these bacteria in the vaginal tract through in vivo studies.

## 5 CONCLUSIONS

The novel combination of *L*. *gasseri* 1A‐TV, *L*. *fermentum* 18A‐TV, and *L*. *crispatus* 35A‐TV characterized both as live strains and as non‐live CFSs in this study showed an antimicrobial activity versus the most common MDR pathogens, such as *E*. *faecium* VRE and KPC‐producing *Klebsiella* *pneumoniae* involved in UTIs, considering the limited antibiotic choice against these MDR microorganisms. In addition, we demonstrated the antimicrobial effect of their cell‐free supernatant, thanks to different substances released by the three *Lactobacilli* both singularly and in combination. The strong bactericidal effect on MDR isolates was also maintained in selected conditions. These results are promising for new vaginal probiotic formulations against MDR bacterial infections.

## CONFLICT OF INTEREST

None declared.

## AUTHOR CONTRIBUTIONS

**Marina Scillato:** Conceptualization (lead); Data curation (equal); Formal analysis (equal); Methodology (lead); Validation (lead); Writing‐original draft (lead); Writing‐review & editing (equal). **Ambra Spitale:** Conceptualization (equal); Data curation (lead); Formal analysis (lead); Methodology (lead); Supervision (lead); Validation (equal); Writing‐original draft (equal); Writing‐review & editing (lead). **Gino Mongelli:** Conceptualization (equal); Data curation (lead); Formal analysis (equal); Methodology (equal); Validation (equal); Writing‐original draft (equal); Writing‐review & editing (equal). **GreteFrancesca Privitera:** Conceptualization (supporting); Methodology (equal); Validation (supporting); Writing‐original draft (equal); Writing‐review & editing (equal). **Katia Mangano:** Methodology (equal); Writing‐original draft (supporting); Writing‐review & editing (equal). **Antonio Cianci:** Investigation (supporting); Writing‐original draft (supporting); Writing‐review & editing (equal). **Stefania Stefani:** Conceptualization (equal); Formal analysis (supporting); Methodology (equal); Project administration (equal); Resources (lead); Supervision (equal); Writing‐original draft (lead); Writing‐review & editing (lead). **Maria Santagati:** Conceptualization (lead); Data curation (lead); Formal analysis (equal); Methodology (lead); Supervision (lead); Validation (lead); Writing‐original draft (lead); Writing‐review & editing (lead).

## ETHICS STATEMENT

Ethical approval is not required. Written informed consent was obtained from all participants.

## Data Availability

All data generated or analyzed during this study are included in this published article.

## 1

**TABLE A1:** Primers designed for the detection of bacteriocin genes

Bacteriocin	Primer name	Primer sequence (5’‐3’)
Nisin A	Nis A	GCGAGCATAATAAACGGCTCTGATT
	Nis A^a^	CAACCACTGAGTATCCAATCTTATACCC
Nisin B	Nis B	CTCAGCTAAATGTTCTAATTGTTGCTTC
	Nis B^a^	AGCTCACAGTATGACTTAACGGGAA
Nisin F	Nis F	TGGAACAGTCTGTGGTTTATTAGGAG
	Nis F^a^	TCACATTCCTCCATGCACAATCTTAA
Gassericin A	GaaA	AGTATCAGTTGGTGGGTTCGTTTG
	GaaA^a^	CACCAACGAGTATTCCAATAAATAGG
Gassericin T	GatA	CACAATAGTGACAGGTCGTAGCACATA
	GatA^a^	CCGTAGCAGCTCCTATTACAGCAT
Gassericin K	MS 480	TCCCCAACTAGTCTATCTGTTGTTCC
	MS 481^a^	GCAATCAGACAGAGTACAGTTACATCTAC
Lactacin F	LaF	AGGGGAATGTGACGATAATGACC
	LaF^a^	TATAGCCAAAATAACCTCCTATTGCTG
Elveticin J	MS 478	TGTATGCGGGCTGGGCTGACT
	MS 479^a^	AGGTTCAGGCTATGGCGATGGAA
Acidocin A	Acd A	CGTAAATTGGGGTAGTGTTGC
	Acd A^a^	AGAACTCAAACGCTGCCTACA
Acidocin B	Acd B	GTCCTGCTTGTGGCTTTGTT
	Acd B^a^	GCCCGTTTGATACAAGTTACCT

^a^ Reverse primers.

**TABLE A2:** In vitro safety assessment of vaginal *Lactobacilli* isolates

Producer Strains	Antibiotic susceptibility profile^a^															Hemolytic activity^b^
	P	AMP	VA	SXT	RD	MTZ	CN	S	TE	C	E	AMC	LEV	CIP	DA	
1A‐TV *L. gasseri*	S	S	S	R	S	R	R	R	S	S	S	S	R	R	S	***−***
18A‐TV *L. fermentum*	S	S	R	R	S	R	R	R	S	S	S	S	R	R	S	***−***
35A‐TV *L. crispatus*	S	S	R	R	S	R	R	R	S	S	S	S	R	R	S	***−***

^a^ Interpretation criteria for the antibiotic susceptibility profile determined by Kirby‐Bauer: resistant (R); susceptible (S). (European Food Safety Authority, 2012). P: Penicillin G, AMP: Ampicillin, VA: Vancomycin, SXT: Cotrimoxazole, RD: Rifampicin, MTZ: Metronidazole, CN: Gentamicin, S: Streptomycin, TE: Tetracycline, C: Chloramphenicol, E: Erythromycin, AMC: Amoxicillin–clavulanic acid, CIP: Ciprofloxacin, LEV: Levofloxacin, DA: Clindamycin

^b^ Interpretation criteria for the hemolytic activity: hemolysis (+); no hemolysis (***−***).
